# The Effect of Thermo-Chemical Treatment on the Water Resistance of Defatted Soybean Flour-Based Wood Adhesive

**DOI:** 10.3390/polym10090955

**Published:** 2018-08-28

**Authors:** Binghan Zhang, Jin Li, Yufei Kan, Jianfang Gao, Yuehong Zhang, Zhenhua Gao

**Affiliations:** 1 College of Material Science and Engineering, Northeast Forestry University, Harbin 150000, China; 18346186030@163.com (B.Z.); zyh6525069@126.com (J.L.); elevenzbh@aliyun.com (Y.K.); shancr@aliyun.com (J.G.); 2 College of Bioresources Chemical and Materials Engineering, Shaanxi University of Science and Technology, Xi’an 710021, China

**Keywords:** defatted soybean flour, thermo-chemical treatment, wood adhesive, sodium dodecyl sulfate (SDS), water resistance

## Abstract

The aim of this study was to effectively improve the water resistance of a defatted soybean flour (DSF)-based adhesive by subjecting DSF to thermo-chemical treatment in the presence of sodium dodecyl sulfate (SDS), and then the crosslinking with epichlorohydrin-modified polyamide (EMPA). The effect of thermo-chemical treatment on the structures and properties of the DSF and DSF-based adhesive were investigated by plywood evaluation, boiling-water-insoluble content, and acetaldehyde value measurements, as well as FTIR, X-ray photoelectron spectroscopic (XPS), X-ray diffraction spectroscopy (XRD), thermogravimetric analysis (TGA), and rheology analyses. The test results revealed that the water resistance of the DSF-based adhesive was significantly improved, attributed to the formation of a solid three-dimensional crosslinked network structure resulted from the repolymerization of DSF, the Maillard reaction between the protein and carbohydrate, and chemical crosslinking between the crosslinker and DSF. Moreover, SDS destroyed the hydrophobic interactions within protein and inhibited macromolecular aggregations during the thermal treatment. Therefore, more reactive groups buried within the globular structure of the soybean protein component of DSF could be released, which supported the repolymerization, Maillard reaction, and chemical crosslinking of DSF, thereby leading to an improved crosslinking density of the cured DSF-based adhesive. In addition, the adhesive composed of thermo-chemically treated DSF and EMPA exhibited preferable viscosity and viscosity stability suitable for the production of wood composites.

## 1. Introduction

Formaldehyde-based adhesives are widely used in the timber industry due to their acceptable water resistance, durable adhesion performance, and low price [1]. However, these adhesives are mainly derived from non-renewable petroleum resources and contain a formaldehyde component that is considered to be a potential human carcinogen [2,3]. Therefore, there is a great demand for the development of safe and environmentally friendly wood adhesives. Defatted soybean flour (DSF), a byproduct of the soybean oil industry, is one of the most promising raw material alternatives for the development of wood adhesives. Thus, DSF is being increasingly used in the wood adhesive industry in China over the past few years due to its abundance, low cost, and renewability. However, the widespread application of traditional DSF-based adhesives is limited due to their poor water resistance and high viscosity [4].

DSF is a complex material comprising approximately 50% protein, 40% soybean carbohydrate, and other minor components [5]. The soybean protein component of DSF has complicated and diverse primary, secondary, tertiary, and quaternary structures, which are mainly built by weak intermolecular interactions, including hydrogen bonds, electrostatic bonds, Van der Waals forces, disulfide bonds, and hydrophobic interactions, leading to low reactivity and poor water resistance [6]. Most of the soybean carbohydrates within DSF are highly hydrophilic polysaccharides that decrease the water resistance of DSF-based adhesives. Many attempts have been made to improve the water resistance of DSF-based adhesives by the modification of DSF, including physical, chemical crosslinking, and biomimetic modification [7,8,9,10,11]. However, most reported modification strategies have focused on the modification of the protein component, which can be divided into two categories. The first category involves protein denaturation using chemical agents or physical means to unfold the protein structure and expose the reactive functional groups (–NH_2_, –COOH, –OH, and –SH). This allows the functional groups to interact with wood, thereby improving the adhesion strength [12,13]. The second category comprises crosslinking modifications by mixing DSF with reactive crosslinkers and/or small synthetic pre-polymers such as glyoxal [14], glutaraldehyde [15], methenamine, genipin [16], polyisocyanates [14,17], modified polyamides [18], epoxy resin [19], phenol-formaldehyde [20], and melamine-urea-formaldehyde [21]. These crosslinkers can react with the reactive groups in DSF and increase the crosslinking density of the corresponding DSF-based adhesive during the hot-press process. On the other hand, the synthetic pre-polymers react with the DSF components to form three-dimensional or interpenetrating network structures that increase the cohesion strength and effectively prevent moisture from intruding into the cured DSF-based adhesives [22]. Some of the above-mentioned modification methods significantly improve the water resistance of DSF-based adhesives. Thus, these adhesives can withstand hydrothermal testing (soaking in water at 63 °C and boiling point). However, studies on the improvement of the water resistance of DSF-based adhesives by modification of the soybean carbohydrate component are scarce [23].

**Figure 1:**
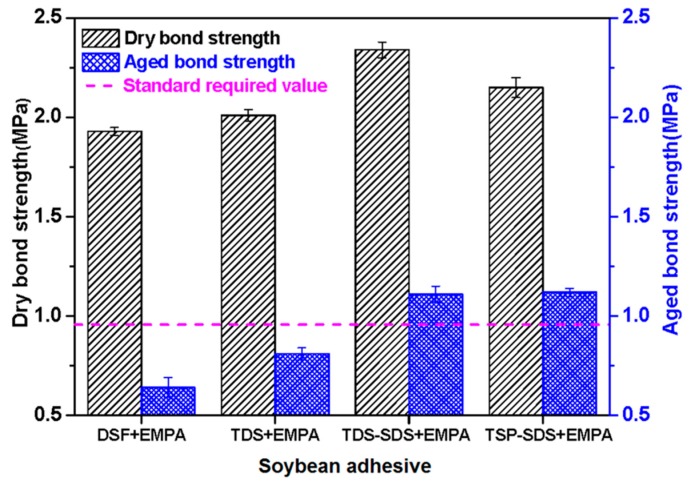
Bond properties of defatted soybean flour (DSF)-and soybean protein isolate (SPI)-based adhesives with various formulations.

A soybean protein isolate (SPI)-based adhesive with good water resistance was recently developed using a combination of activation and chemical crosslinking modifications [6,24]. Activation modification was carried out by thermo-chemical treatment of the SPI at 120 °C for 0.5 h in the presence of protein-denaturing agents such as hydrochloric acid, sodium sulfite, SDS, and their combination. These denaturing agents unfolded and stretched the compact globular structure of the SPI by destroying its hydrogen and disulfide bonds, and its hydrophobic moieties. This was beneficial to the repolymerization of the SPI molecules and the exposure of the reactive groups buried in the globular structure of the SPI after thermo-chemical treatment. Thus, the thermo-chemically treated SPI could be crosslinked more sufficiently in the subsequent chemical crosslinking modification by epichlorohydrin-modified polyamide (EMPA) to form three-dimensional crosslinked network structures and convert the hydrophilic groups into hydrophobic structures. Consequently, the combination of SPI repolymerization after thermo-chemical treatment and the increased crosslinking efficiency between the SPI and crosslinker led to a significant improvement in the water resistance of the resultant adhesive [24], which could withstand a 28 h boiling-dry-boiling hydrothermal treatment and maintain a hydrothermal-aged wet bond strength up to 1.22 MPa (exceeding the required value of 0.98 MPa for structural use), as presented in Figure 1.

However, its high cost has made SPI less attractive as a starting material for the preparation of wood adhesive. DSF, a by-product of the soybean oil industry that is mainly used as animal feed with low added value, costs a fraction (1/4 to 1/5) of the cost of SPI [25]. Thus, it is considered one of the most promising candidates for the preparation of wood adhesives. To develop a low-cost soybean-based adhesive with the desired water resistance using DSF, this work investigated the effect of thermo-chemical treatment on the properties of DSF and DSF-based adhesives. Notably, the Maillard reaction is a complex set of reactions that takes place between proteins and carbonyl compounds including soybean carbohydrate at high temperatures [5]. Thus, the interactions between the soybean carbohydrate and protein component within DSF during thermo-chemical treatment were also investigated [23,26]. Therefore, changes in the functional groups, secondary structure, water solubility, reactivity, crystallinity, and thermo-stability of DSF, before and after thermo-chemical treatment, were evaluated to elucidate the improvement mechanism of the water resistance of DSF-based adhesives.

## 2. Materials and Methods

### 2.1. Materials

DSF, with an average protein content of 53.4%, and SPI, with a protein content of 93.4 wt %, (particle size < 0.096 mm) were purchased from Harbin High-Tech Soybean Food Co., Ltd., Harbin, China. EMPA aqueous solution was synthesized in our lab using diethylenetriamine, adipic acid, and epichlorohydrin, which had a solid content of 13.8%, pH of 2.6, and a viscosity of 96.4 mPa.s (25 °C). Diethylenetriamine, adipic acid, and epichlorohydrin were obtained from local chemical companies, Harbin, China. Reagent-grade SDS (with a content of 99.5%) was purchased from Tianjin Kermel Chemical Reagent Co., Ltd., Tianjin, China. Birch veneers, with dimensions of 420 mm × 420 mm × 1.6 mm and moisture content of 8–10%, were supplied by a local plywood plant in Harbin.

### 2.2. Thermo-Chemical Treatment of DSF

About 100 g DSF was blended with 35 g of a solution containing 2.5 g SDS in a high-speed mixer at 800 rpm. After uniform mixing, the mixture was placed on a polytetrafluoroethylene film to form a thin layer. This was then placed in an oven preheated at 140 °C for 30 min. After cooling to room temperature, the product was ground to a fine powder, passed through a 120-mesh sieve, and marked as TDS-SDS. SPI was also thermo-chemically treated as that of TDS-SDS by just replacing DSF by SPI (labeled TSP-SDS). Native DSF and DSF that were thermally treated without using SDS were selected as the controls and labeled DSF and TDS, respectively.

### 2.3. Preparation of the DSF-Based Adhesives and Cured Samples

The DSF-based adhesives were prepared by mechanically blending 40 parts soybean flour powder (TDS-SDS, DSF, and TDS) with 100 parts liquid EMPA solution by mass, at room temperature, until no particle clusters were observed. These samples were labeled “TDS-SDS+EMPA”, “DSF+EMPA”, and “TDS+EMPA”, respectively.

About 5 g of the above prepared DSF-based adhesives were then placed in a polytetrafluoroethylene container and cured at 120–125 °C for 2 h to produce the cured DSF-based adhesive samples (with curing reactions illustrated in Figure 3). After cooling to room temperature, these samples were ground to a fine powder and passed through a 160-mesh sieve prior to characterization.

### 2.4. Bond Strength and Water Resistance by Plywood Evaluation

Three-ply plywood panels were fabricated with liquid adhesive loadings of 180 g/m^2^ (single glue line) by coating the adhesive onto both faces of the core veneer. Two face veneers without adhesive and an adhesive-coated veneer were stacked by hand with their grains vertical to each other. The stacks were then cold pressed at 1.1 MPa and room temperature for 45 min and finally hot pressed at 120 °C and 1.3 MPa for 4 min. After hot pressing, the panels were stored under a 20–25 °C and 40–60 RM% chamber for ≥24 h prior to testing. A total of 40 specimens with a glued area of 25 mm × 25 mm were cut according to the commercial standard JIS K6806-2003 to determine the bond strength (20 specimens) and water resistance (or bond durability, 20 specimens), in terms of the dry strength and aged wet strength, respectively, by a tensile shear mode. The test was performed using a tensile testing machine with a crosshead speed of 5 mm/min. The specimens for the aged wet strength test underwent a 28 h boiling-dry-boiling treatment (4 h of boiling, 20 h of oven-drying at 63 ± 2 °C, and 4 h of boiling), were cooled to room temperature, and subsequently measured under wet state [27]. In contrast, the specimens for the dry bond strength tests were measured under dry state.

### 2.5. Water-Insoluble Content Determination of DSF, TDS, TDS-SDS, and Their Corresponding Cured Adhesives

Approximately 2.0 g sample (DSF, TDS, TDS-SDS, and their corresponding cured adhesives; passed through a 160-mesh sieve, *W*_0_, accurate to 0.0001 g), and 200.0 g distilled water were added in a 250 mL flask equipped with mechanical stirring and refluxing equipment. The mixture was stirred and boiled for 4 h. The afforded dispersion was cooled to room temperature and filtered, and the collected residue was rinsed twice with 50 mL distilled water. The glass-filter paper was dried to constant weight (W_1_, accurate to 0.0001 g) in advance. The glass-filter paper including filtered residue was oven-dried at 120 °C to constant weight (*W*_2_, accurate to 0.0001 g). The boiling-water-insoluble content was defined as the mass percentage of test sample insoluble in boiling water and calculated as [(*W*_2_ − *W*_1_)/*W*_0_] × 100%.

### 2.6. Acetaldehyde Value of DSF, TDS, and TDS-SDS

A 100 mL reaction flask equipped with a mechanical stirrer, thermostat, and condenser (Lichen, Shanghai, China) for reflux was charged with approximately 1.5 g sample (DSF, TDS, and TDS-SDS; particle size, passed through a 160-mesh sieve; *m*_1_, accurate to 0.0001 g), 50.0 mL water, and 5.0 mL of 40 wt % acetaldehyde. The pH of the mixture was adjusted to 8.5–8.7 using a 20 wt % sodium hydroxide solution. The mixture was then maintained at 50 ± 2 °C for 120 min, subsequently allowed to cool to room temperature, and then filtered. The residue was rinsed twice with 50 mL distilled water. All the filtered solutions, including the rinsed solution, were diluted to 1000 mL in a volumetric flask. The unreacted acetaldehyde content in the diluted filtrate (*F*_1_, mmol/L) was tested according to the test method for free formaldehyde described in the literature [24]. The test was repeated in triplicate. Three blank tests were also conducted under the same conditions, without the addition of DSF, to determine the total free acetaldehyde content (*F*_0_, mmol/L) prior to the reaction. The acetaldehyde value (mg/g) of the sample was defined as the equivalent mass of acetaldehyde (mg) that can react with 1 g solid DSF, calculated as [(*F*_0_ − *F*_1_) × 44]/*m*_1_. 

### 2.7. X-ray Photoelectron Spectroscopic (XPS) Analysis

The surface chemical compositions of the native DSF, TDS, and TDS-SDS samples were analyzed using a Kα X-ray photoelectron spectrometer (Thermo Fisher Scientific Inc., Waltham, MA, USA) with monochromatic Al-Kα radiation at 100 W. High-energy photoemission spectra (Thermo Fisher Scientific Inc., Waltham, MA, USA) were recorded using a pass energy of 50 eV and a resolution of 0.1 eV. Curve-fitting analysis of the C_1s_ peak was performed using the Gaussian-Lorentzian curve fitting function to result in the smallest least-square coefficient by XPS Peak 4.0 software.

### 2.8. FT-IR Analysis

Structural changes in the DSF, TDS, and TDS-SDS were observed using Fourier transform infrared (FTIR) spectroscopy (Nicolet Co., WI, USA; 4000–400 cm^−1^; 4 cm^−1^ resolution; 32 scans). The peak decomposition was performed in the region 1700–1600 cm^−1^ assigned to the amide I band of the soybean protein component. A second-derivative analysis was carried out to attain quantitative information about the secondary structures of the protein in DSF. Thus, after baseline correction, the Fourier self-deconvolution and the deconvoluted (differential) spectra were first resolved. Subsequently, the individual component bands were quantified according to a Gaussian curve fit (GCF). The procedure maintained the initial band positions at an interval of 4 ± 1 cm^−1^, excluding bands with negative heights. It also retained the bandwidth within the expected limits in agreement with the theoretical boundaries and predictions. The relative amounts of the various DSF secondary structures were determined from the second derivative of the amide I band (areas under the bands assigned to a particular substructure). The difference between the measured spectrum and the curve fit was calculated as an internal control of the success of the curve fitting process.

### 2.9. X-ray Diffraction Spectroscopy (XRD) Analysis

The changes in crystalline structure of the native DSF, TDS, and TDS-SDS treated at various temperatures were recorded on a D/MAX-2200 diffractometer (Rigaku, Tokyo, Japan) using a Cu-Kα source. Diffraction data were collected from 5° to 50° with a step interval of 0.02°, an accelerating voltage of 40 kV, and a current of 30 mA.

### 2.10. Thermogravimetric Analysis (TGA)

Thermogravimetric analysis was performed on a TA Instruments Discovery (NETZSCH Co., Selb, Germany) TGA. All samples were tested from room temperature to 800 °C at a scanning rate of 10 °C/min under nitrogen (25 mL/min).

### 2.11. Dynamic Viscoelastic Measurements

The apparent viscosity of the fresh DSF-based adhesives (“TDS-SDS+EMPA”, “DSF+EMPA”, and “TDS+EMPA”) was determined using an AR 2000ex rheometer (TA Instruments Ltd., New Castle, DE, USA) with a parallel plate fixture (40 mm diameter with thermostat). The distance was set to 1.5 mm for all the measurements. The experiments were conducted under a steady shear flow at 25 °C using two modes: A frequency mode (shear rate range = 0.1–600 s^−1^ in 10 s^−1^ increments), and a time mode that maintained the sample at a shear rate of 10 s^−1^ for 40 min.

### 2.12. Statistical Analysis

The data in the current study were statistically evaluated using the statistical software package Minitab version 15 and reported as the mean value ± standard deviation of the replicates. A single-factor analysis of variance was conducted to identify significant differences among mean values according to least significant difference criteria with a 95% confidence level (*p* < 0.05).

## 3. Results

The bond strength and water resistance of the DSF-based adhesives are presented in Figure 1. The plywood bonded with the “DSF+EMPA” adhesive (formulated by native DSF and EMPA as the crosslinker) could withstand a 28 h boiling-dry-boiling hydrothermal test; however, the aged wet bond strength could not meet the required value (0.98 MPa) for structural use according to the commercial standard JIS K6806-2003. This was mainly attributed to the most reactive groups (–NH_2_, –OH, and –COOH) of the DSF protein component being buried within the highly ordered and compact globular structure. This leads to insufficient contact area with the wood surface, an undesired protein-wood interface, and limited reactive sites for potential adsorption interactions and chemical reactions. As expected, the plywood bonded with the “TDS-SDS+EMPA” adhesive, formulated by thermo-chemically treated DSF in the presence of SDS, exhibited superior water resistance over that of the control adhesive “DSF+EMPA.” This was confirmed by the 73% increase in the aged wet bond strength of the “TDS-SDS+EMPA” adhesive over that of the control, which is almost the same as that of the “TSP-SDS+EMPA” adhesive (TSP-SDS refers to thermo-chemically treated SPI in the presence of SDS). These results indicated that similar to SPI, DSF could also be effectively activated by thermo-chemical treatment. In contrast, the “TDS+EMPA” adhesive, composed of sole thermo-chemically treated DSF without SDS, displayed a less improvement in water resistance than that observed for the “TDS-SDS+EMPA” adhesive. Moreover, the aged wet bond strength was only 26% higher than that of the control adhesive “DSF+EMPA.” This confirmed that compared to sole thermal treatment, the thermo-chemical treatment of DSF in the presence of SDS could improve the water resistance of DSF-based adhesives more effectively.

**Figure 2:**
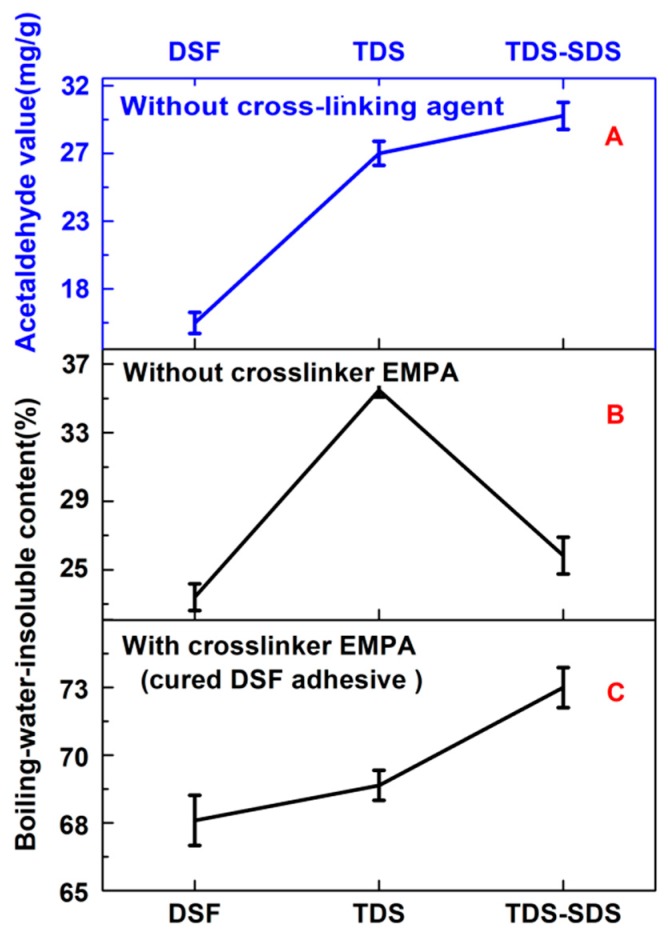
(**A**) Acetaldehyde values and (**B**,**C**) boiling-water-insoluble contents of various DSF samples.

Thermal treatment is often used to dissociate proteins into their constituent subunits, unfold their structure and surface, and therefore expose their hydrophilic groups to reactivity [28]. However, the high-temperature thermal treatment of DSF was usually accompanied by an aggregation of unfolded protein molecules [29]. Thus, some protein reactive groups were re-embedded, thereby leading to less crosslinking reactions between the protein and crosslinker. Consequently, the plywood bonded with the “TDS+EMPA” adhesive exhibited lower aged wet bond strength than that with the “TDS-SDS+EMPA” adhesive (Figure 1). This was attributed to a lower degree of crosslinking (Figure 2C) caused by less available reactive groups/sites (Figure 2A) compared to those present in the TDS-SDS sample.

SDS is a disperser that can unfold the globular structure of soybean proteins by destroying the hydrophobic moieties and exposing the buried reactive groups [13]. Moreover, it can bind strongly with the protein molecules through hydrophobic interactions. This increases the surface charge and intermolecular repulsion of the protein and therefore inhibits insoluble macromolecular aggregation [30]. As a result, the thermo-chemically treated TDS-SDS sample displayed a lower boiling-water-insoluble content (25%) than that of the thermally treated TDS sample (35%). However, both values were higher than that observed for the native DSF (22%, Figure 2B). These results indicated that both the thermal and thermo-chemical treatments could lead to the repolymerization of the DSF components via the –SH and S–S interchange reactions and the Maillard reaction between the protein and carbohydrate at 140 °C. Additionally, the presence of SDS could effectively inhibit macromolecular aggregation during thermal treatment.

**Figure 3:**
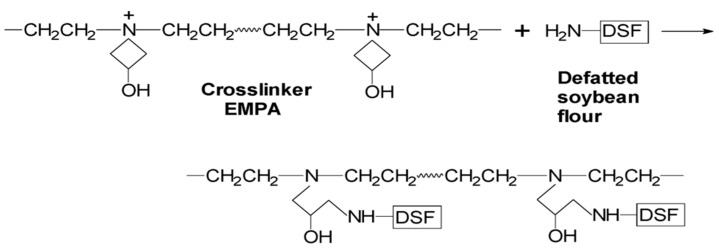
Schematic illustration of the crosslinking reaction between DSF and epichlorohydrin-modified polyamide (EMPA).

The results presented in Figure 2C reveal that cured DSF based adhesives composed of TDS-SDS displayed a higher boiling-water-insoluble content (72%) than that of the adhesive solely composed of TDS (69%), suggesting that the “TDS-SDS+EMPA” adhesive had a higher crosslinking density than the “TDS+EMPA” adhesive. This was attributed to the release of more reactive groups, such as amino groups (–NH_2_) after the DSF was thermo-chemically treated. These groups can react with the azetidinium groups of the EMPA crosslinker [31,32] (Figure 3), resulting in the formation of a dense and crosslinked network structure that prevents the invasion of water. The acetaldehyde value test in Figure 2A confirmed that more reactive amino groups were released after both thermal and thermo-chemical treatment. Because the consumption of acetaldehyde in the DSF-acetaldehyde system was attributed to the reactions between the acetaldehyde and amino groups of the soybean protein, the acetaldehyde value represented the number of amino groups that were able to react with the crosslinker in various DSF samples. A high acetaldehyde value suggested the presence of more crosslinking reactions between the DSF sample and the post-added EMPA crosslinker.

**Figure 4:**
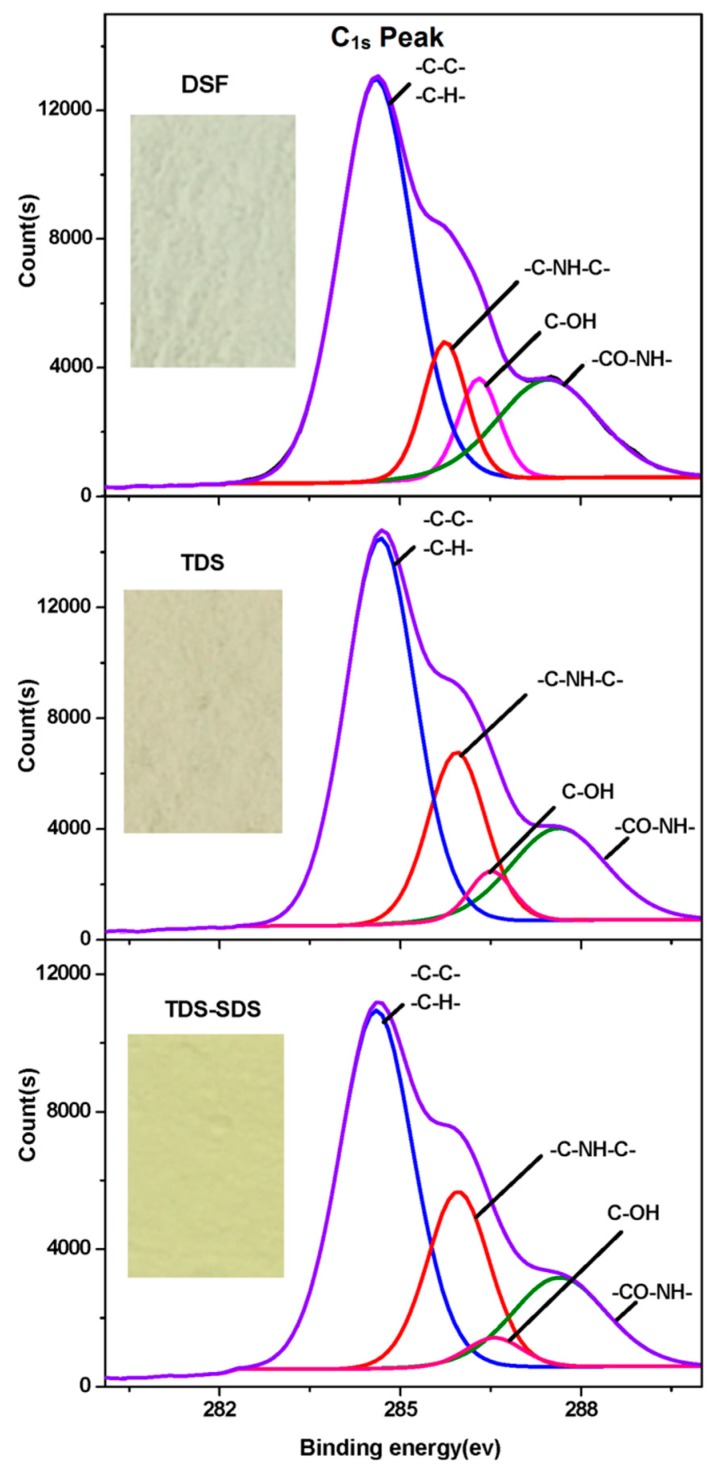
X-ray photoelectron spectroscopic (XPS) spectra and photographs of various DSF samples.

**Figure 5:**
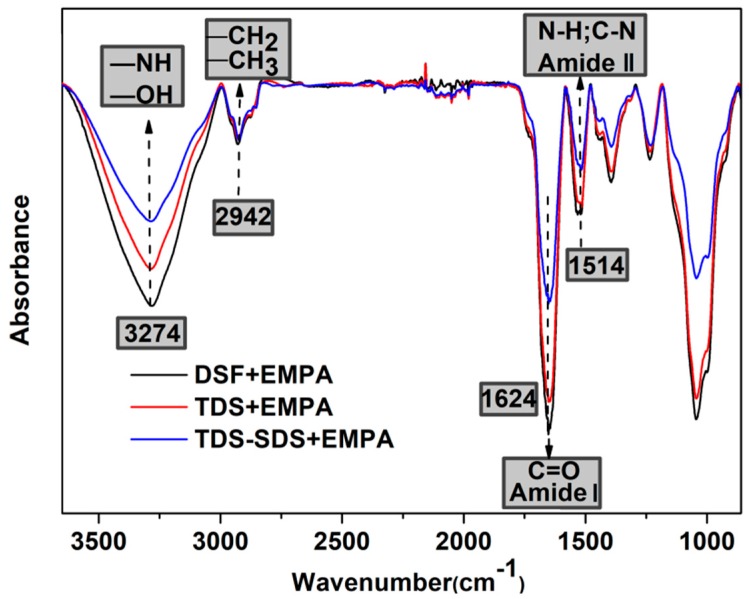
FT-IR spectra of various DSF-based adhesives.

The optical photographs in Figure 4 illustrates that the colors of the DSF samples changed from grey to yellowish grey and light yellow after thermal and thermo-chemical treatment, respectively, indicating the occurrence of the Maillard reaction between the protein and the soybean carbohydrates. The Maillard reaction of DSF is related to a range of complex browning reactions between the protein amino groups and the carbohydrate carbonyl groups (such as aldehyde groups) upon heating. This reaction is generally accompanied by the consumption of amino groups and the formation of Amadori compounds (–C–O–), Schiff bases (–C=N–), and pyrazines (–C–N–) [33]. However, all the structures involved in the Maillard reaction were difficult to observe with FT-IR analysis (Figure 5) due to the overlap of their peaks in the fingerprint. No new IR absorption peaks were detected in the FT-IR spectra; however, the peak intensities at 1624 cm^−1^ (amide I, C=O stretching) and 1514 cm^−1^ (amide II, N–H bending) decreased. This indicated a change in the concentration of these groups. These data confirmed that repolymerization of the soybean protein and the Maillard reaction between the protein and soybean carbohydrate within DSF occurred during the thermal and thermo-chemical treatment of DSF.

In XPS analysis, the C1s photoelectron peaks of DSF, TDS, and TDS-SDS were deconvoluted, according to results reported in the literature [34,35]: Peak C1 at 284.6 eV, corresponding to C–C or C–H bonds, indicates the presence of hydrophobic properties; peak C2 at 285.7 eV, corresponding to C–NH–C, results from the reaction between the carbohydrate and protein; peak C3 at 286.4 eV, related to C–OH which was derived from the carbohydrate, indicates the presence of hydrophilic properties; and peak C4 at 287.8 eV, assigned to –CO–NH–, results from the condensation reaction between the primary amine and carboxyl groups. The XPS data in Figure 4 and Table 1 reveal an increase in the areas of peaks C2 and C4 and the significant decrease in the area of Peak C3 after DSF underwent thermal and thermo-chemical treatment. These results indicate that a Maillard reaction occurred between the soybean protein and carbohydrate components. Because SDS favors the release of reactive amino groups during thermo-chemical treatment, TDS-SDS was more reactive and further supported the Maillard reaction between carbohydrates and protein, as confirmed by the marked decrease in the NH_2_ and C=O IR absorption peaks in Figure 5. As the second largest component in DSF, soybean carbohydrates exhibited negative effects on the bond strength and water resistance of the adhesive. This was attributed to the increased water absorption due to the presence of abundant hydrophilic hydroxyl groups [24]. These data suggest that sufficient carbohydrate crosslinking via thermo-chemical treatment could effectively improve the water resistance of DSF-based adhesives. Moreover, these results are comparable to the results observed for the SPI-based adhesive (Figure 1).

**Figure 6:**
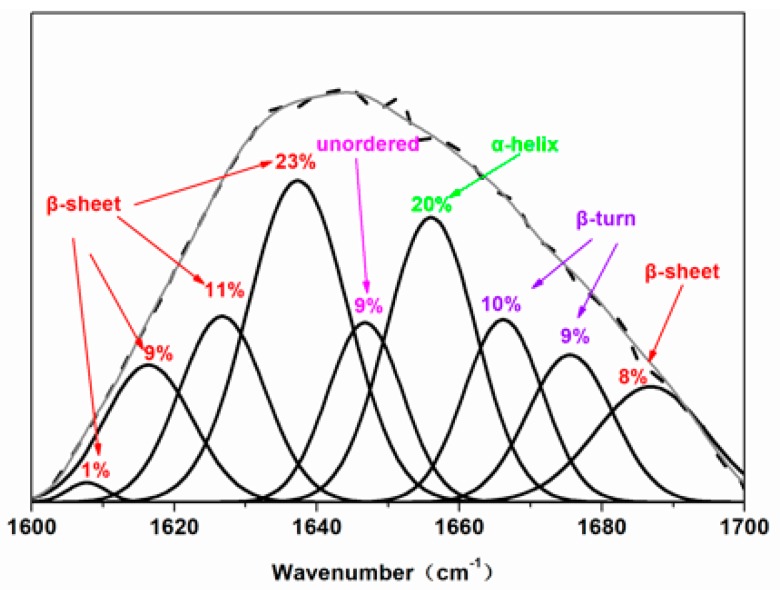
Deconvolution of the amide I spectra (continuous curve), the Gaussian curve fit (GCF) bands thereof (point line), and the second-derivative spectra of the TDS-sodium dodecyl sulfate (SDS) sample.

**Figure 7:**
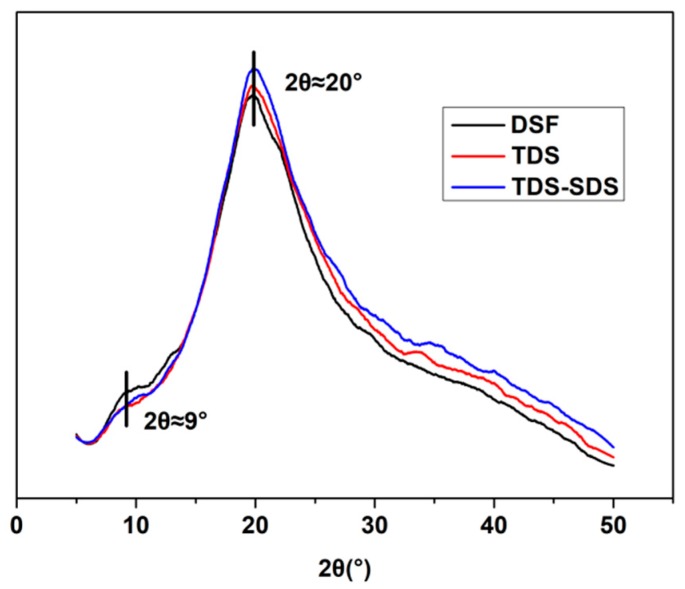
X-ray Diffraction Spectroscopy (XRD) patterns of various DSF samples.

The IR peak deconvolution of amide I (C=O stretching vibration) is presented in Figure 6. The areas of the assigned amide I bands in the second derivative spectra linearly correspond to the amount of the different types of secondary structures present in the protein [36]. The corresponding relationship between each sub-peak and the secondary structure were assigned as follows: The bands corresponding to the β-sheet appeared in the frequency regions 1610–1640 cm^−1^ and 1670–1690 cm^−1^, the β-turn appeared in the region 1660–1670 cm^-1^, the random coil structure appeared in the region 1640–1650 cm^−1^, and the α-helix appeared in the region 1650–1660 cm^−1^ [36,37,38]. The α-helix, β-turn, β-sheet, and unordered structure contents in DSF, TDS, and TDS-SDS are listed in Table 2. After thermal and thermo-chemical treatment, the β-sheet structure contents increased from 4% to 31% and 52%, respectively, in accordance with the XRD results. The XRD peaks at 2θ of 9° and 20° belong to the α-helix and β-sheet structures of the DSF soybean protein component, respectively (Figure 7). Compared to the values observed for DSF and TDS, the peak intensity of TDS-SDS at 2θ ≈ 9° decreased, whereas that at 2θ ≈ 20° increased. The β-sheet structure is an important form of protein secondary structure. An increased β-sheet content led to more regular peptide chains, attributed to the rearrangement of the hydrophobic chains formed in the hydrophobic regions. This indicated that the rearrangement of the protein molecules led to more regular protein structures in DSF after thermal and thermo-chemical treatment.

**Figure 8:**
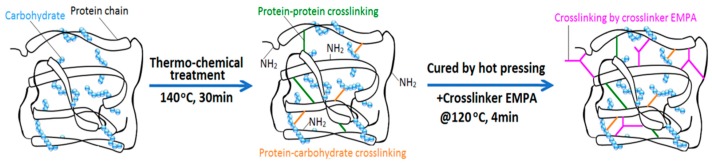
Schematic illustration of the crosslinking modes of TDS-SDS and its cured adhesive.

**Figure 9:**
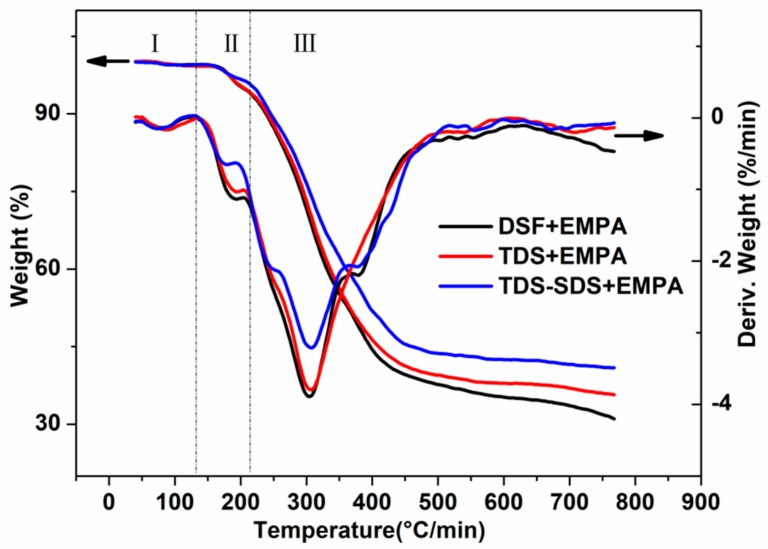
Thermogravimetric analysis (TGA) and DTG curves of various DSF-based adhesives.

The repolymerization of the proteins, rearrangement of the protein hydrophobic chains, Maillard reaction between the protein and carbohydrate, and effective crosslinking reaction between TDS-SDS and EMPA led to the formation of a more compact network structure, as illustrated in Figure 8. Thus, all these factors contributed towards the improvement in the water resistance of the cured adhesive (TDS-SDS+EMPA). The TGA data in Figure 9 revealed that there are no significant differences in the main decomposition temperatures of the various cured adhesives. However, the adhesive “TDS-SDS+EMPA” displayed a slower and lower weight loss, indicating its improved thermal stability. This was attributed to the improved crosslinking density and more compact network structure of the cured adhesive.

**Figure 10:**
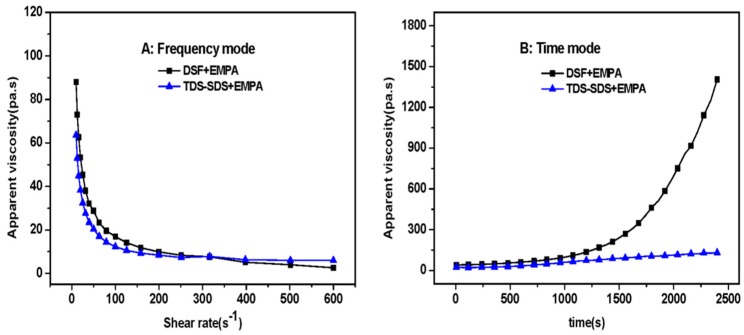
Apparent viscosity of various DSF-based adhesives, frequency mode (**A**) and Time mode (**B**).

Due to the higher protein content and large molecular weight of soybean protein, the TSP-SDS+EMPA adhesive composed of thermo-chemically treated SPI in the presence of SDS (TSP-SDS) and EMPA as the crosslinker (37 wt % solid content) displayed a viscosity between 6.86 × 10^4^ and 7.02 × 10^4^ mPa.s (BROOKFIELD DV-II+PRO viscometer at 25 °C and 10 s^−1^ rotating speed). Thus, it was difficult to spread this SPI adhesive evenly onto the veneer. Moreover, the higher viscosity displayed negative effects on the wettability and penetration of the adhesive into the wood substrate. On the other hand, the viscosity of the DSF-based adhesive composed of TDS-SDS with the same solid content was much lower (~1.13 × 10^4^ mPa.s, by BROOKFIELD DV-II+PRO viscometer at 25 °C and 10 s^−1^ rotating speed). Therefore, while displaying the improved bond strength and water resistance observed for the SPI-based adhesive, the DSF-based adhesive was easier to spread and supported wettability and penetration into the wood substrate (Figure 1). The apparent viscosities of the two DSF-based adhesives, determined by rheological testing, are presented in Figure 10.

In frequency mode, the apparent viscosities (Figure 10A) decreased as the shear rate increased from 0.1 to 300 s^−1^, revealing that the shear-thinning behaviors of the two DSF-based adhesives were similar to that of other reported soybean adhesives [39]. Although they displayed a similar decreasing tendency, the initial apparent viscosity of the “DSF+EMPA” adhesive was 38% greater than that of the “TDS-SDS+EMPA” adhesive. This increase indicated that in the EMPA solution, DSF disperses and then unfolds more easily than TDS-SDS. This leads to more molecular entanglement because DSF has no such crosslinked structures as TDS-SDS resulted from the protein re-polymerization and protein-carbohydrate Maillard reaction during thermo-chemical treatment. These results were also confirmed by the apparent viscosities measured in time mode (Figure 10B). Thus, the “TDS-SDS+EMPA” adhesive displayed a slow linear increase in viscosity, from 87 to 131 Pa.s, while the “DSF+EMPA” adhesive exhibited a sharp exponential increase, from 2101 to 1404 Pa.s, at the same shear rate (10 s^−1^) at 25 °C for 40 min. The shear rate in the time-mode test was selected because it is comparable to the common roller rotating rate (400–800 rpm) of an adhesive spreader in commercial plywood production. Because the adhesive load on the veneer is highly correlated to the adhesive viscosity, the slow increase in apparent viscosity indicated that the “TDS-SDS+EMPA” adhesive displayed better viscosity stability than the “DSF+EMPA” adhesive. Thus, the former adhesive was more beneficial to the stabilization of plywood production.

## 4. Conclusions

A DSF-based adhesive with good water resistance and low viscosity was developed, which comprised thermo-chemical treatment of DSF in the presence of SDS at 140 °C followed by crosslinking modification with EMPA. Because of the destruction of hydrophobic moieties and inhibition of macromolecular aggregation by SDS, the thermo-chemically treated DSF released more reactive groups. This aided protein repolymerization, rearrangement of the protein hydrophobic chains, the Maillard reaction between the protein and soybean carbohydrate, and the effective crosslinking reaction between DSF and EMPA. The formation of a more compact network structure led to the effective improvement in the water resistance of the “TDS-SDS+EMPA” adhesive. Thus, the proposed adhesive could withstand a 28 h boiling-dry-boiling hydrothermal treatment and maintain a wet-bond strength of 1.21 MPa, exceeding the required value for structural use (0.98 MPa). However, the Maillard reaction during the thermo-chemical treatment of DSF is to be further evaluated delicately in order to effectively improve and/or tailor the bond performance of DSF-based adhesives. 

## Figures and Tables

**Table 1:** Structure contents of the defatted soybean flour (DSF), TDS, and TDS-sodium dodecyl sulfate (SDS) samples by X-ray photoelectron spectroscopic (XPS) analysis.

Samples	C–C (%)	C–NH–C (%)	C–OH (%)	NH–CO (%)
DSF	59	12	8	20
TDS	58	18	4	19
TDS-SDS	56	23	3	17

**Table 2:** Secondary structure content in the DSF, TDS, and TDS-SDS samples.

Samples	β-Sheet (%)	Unordered (%)	α-Helix (%)	β-Turn (%)
DSF	4	46	37	14
TDS	31	31	10	30
TDS-SDS	52	9	20	19
